# Rapid Analysis of Milk Using Low-Cost Pocket-Size NIR Spectrometers and Multivariate Analysis

**DOI:** 10.3390/foods9081090

**Published:** 2020-08-10

**Authors:** Jordi Riu, Giulia Gorla, Dib Chakif, Ricard Boqué, Barbara Giussani

**Affiliations:** 1 Department of Analytical Chemistry and Organic Chemistry, Universitat Rovira i Virgili, 43007 Tarragona, Spain; jordi.riu@urv.cat (J.R.); dib.chakif@hotmail.com (D.C.); ricard.boque@urv.cat (R.B.); 2 Dipartimento di Scienza e Alta Tecnologia, Università degli Studi dell’Insubria, Via Valleggio, 9, 22100 Como, Italy; ggorla@uninsubria.it

**Keywords:** NIR, milk, portable instrumentation, spectroscopy, green analytical chemistry, multivariate analysis, classification

## Abstract

The miniaturisation of analytical devices, reduction of analytical data acquisition time, or the reduction of waste generation throughout the analytical process are important requirements of modern analytical chemistry, and in particular of green analytical chemistry. Green analytical chemistry has fostered the development of a new generation of miniaturized near-infrared spectroscopy (NIR) spectrometric systems. However, one of the drawbacks of these systems is the need for a compromise between the performance parameters (accuracy and sensitivity) and the aforementioned requirements of green analytical chemistry. In this paper, we evaluated the capabilities of two recently developed portable NIR instruments (SCiO and NeoSpectra) to achieve a rapid, simple and low-cost quantitative determination of commercial milk macronutrients. Commercial milk samples from Italy, Switzerland and Spain were chosen, covering the maximum range of variability in protein, carbohydrate and fat content, and multivariate calibration was used to correlate the recorded spectra with the macronutrient content of milk. Both SCiO and NeoSpectra can provide a fast and reliable analysis of fats in commercial milk, and they are able to correctly classify milk according to fat level. SCiO can also provide predictions of protein content and classification according to presence or absence of lactose.

## 1. Introduction

Cow’s milk is an important component of the human diet, with an estimated worldwide production of approximately 800 million tonnes in 2016 and growth expectations of 981 million tonnes in 2028 [1]. From an economic point of view, around 150 million farms worldwide are involved in milk production. The composition of the milk largely determines its nutritional value and provides information on the health status of the cow. Animal health monitoring increases livestock management in dairy production and helps the producer to optimize animal management while reducing the workload [2]. 

Due to its importance, milk has been analysed for decades and the standard methods of analysis in many cases are old and tedious. For instance, a common method for the determination of fat is the Gerber method (patented in 1891), which separates fats by adding sulphuric acid and then reads the fat content using a calibrated butyrometer [3], and the reference method for the analysis of fats is a gravimetric method [4]. Despite being reference methods, these classical methods of analysis are nowadays less frequently used. Instrumental techniques of analysis are also widely used in milk analysis, and among them, chromatographic techniques are extensively used [5]. 

However, most methods typically involve multiple steps, such as an extraction procedure, methylation, methyl ester extraction and gas chromatographic analysis for the determination of fatty acids, and similar procedures for other compounds [6,7,8,9,10]. Therefore, due to the need for laboratory-based equipment, these techniques do not allow the analysis of milk on-site, making it necessary to move the sample from the farm to the lab. This makes it difficult to establish rapid quality controls to make decisions when it comes to improve milk quality continuously and quickly. Nowadays, the goals of greener analytical chemistry are to use methods that cost as little as possible in terms of sample pre-treatment and are applicable online or in situ to avoid wasting time, reagents, and money [11].

An alternative to classical and chromatographic techniques are spectroscopic techniques in combination with chemometric analysis, with the advantage that they allow different parameters to be determined at the same time. IR spectroscopy was used to detect milk adulteration [12] and to reliably determine milk content ([13,14,15] and references therein). In addition, more in-depth analyses have been proposed; e.g., Soyeurt et al. [16] were able to determine the fatty acid content in milk with mid-infrared spectroscopy (MIR) and Coppa et al. [17] optimized a method with the same aim based on near-infrared spectroscopy (NIR). In 2014, a comparison between NIR and MIR was also proposed [18]. 

Undoubtedly, benchtop NIR instruments together with chemometric methods of data analysis can provide robust results with a high degree of accuracy in a simple way [19]. However, these instruments are expensive and sometimes samples need pre-treatment or have to be transferred to the laboratory. In recent years, spectroscopic instrumentation has made significant advances and made low-cost miniaturized infrared instrumentation available [20,21]. These advances have led to instruments based on MEMS (micro-electro-mechanical systems) and MEOMS (micro-opto-electro-mechanical systems), which offer significant advantages in terms of price, size or weight [22,23]. All of these advances, combined with chemometric analysis in the cloud and integration with smartphones [24,25] pave the way for decentralized and immediate on-farm analysis. 

Various commercial hand-held devices, such as the Polychromix PHAZIR^™^ (Polychromix Inc., Wilmington, MA, USA) [26,27], 4200 FlexScan FTIR (Agilent Technologies Inc., Danbury, CT, USA) [28], Micro-NIR 1700 (JDSU, Milpitas, CA, USA) [29,30] or the Agilent 4500 portable ATR-FTIR (Agilent Technologies, Inc., Santa Clara, CA, USA) [31] have been used for milk content analysis. Despite being portable and small in size, all hand-held devices still have prices around EUR 15,000–20,000, which hampers their distribution in all types of facilities and situations. SCiO (Consumer Physics, Herzliya, Israel) and NeoSpectra (Si-Ware, Cairo, Egypt), with prices between EUR 950–2500, are two alternatives for the low-cost decentralized measurement of milk and they are even affordable for online measurements or quality control monitoring in small facilities.

The goal of this paper was to analyse whether these two pocket-sized NIR spectrophotometers, SCiO and NeoSpectra, can be used in the rapid and low-cost analysis of milk without performing any pre-treatment or using any specific measuring head. To meet these objectives, 45 commercial milks were analysed with these instruments and the contents of the main nutritional components were predicted. Due to the light scattering in milk and the nature of the spectroscopic signals (reflectance), we will expect better results in predicting the fat and protein content, and poorer results for carbohydrates, which are small molecules dissolved and dispersed in the milk matrix that badly contribute to the scattering of light.

## 2. Instruments and Samples

### 2.1. Instrumentation

SCiO (Consumer Physics, Herzliya, Israel) and the NeoSpectra (Si-Ware, Cairo, Egypt) devices were used for the milk analysis.

SCiO consists of an Osram broadband IR led coupled to a dispersive element and a silicon-based 1.2 Mpixel CMOS image sensor. The dimensions of the device are 67.7 × 40.2 × 18.8 mm with a weight of 35 g, and it can operate at temperatures ranging from 4 to 35 °C. The device is controlled by the Android/iOS ‘The Lab’ app and Bluetooth is used to communicate with a smartphone. SCiO has to be calibrated each time the app is started. The calibration process is very fast when using the app and a calibration standard is included in the cover of the instrument. One of the advantages of the instrument is that the warm-up time is null (the producer declared it, and we observed that the spectra were stable from the very beginning of the analysis). The SCiO wavelength range is from 740 to 1070 nm, which means that the SCiO also reaches the visible part of the electromagnetic spectrum, with a declared resolution of 1 nm (an average resolution of 13 cm^−1^ is also reported [32], while some authors claim that the real resolution is lower [21]). The acquisition time is around 2–5 s and no experimental parameters can be set (e.g., the scan time). Liquid samples can be scanned at a distance of up to 10 mm, while for solid samples SCiO can also perform contact measurements. The scattered and emitted light from the sample first passes through an optical filter and is then dispersed by a fast Fourier transform focusing element into an image sensor. The app allows the user to scan any material and save the spectra in the cloud. In the private area of the website (SCiO Lab, thelab.consumerphysics.com), the user can directly perform a limited number of pre-treatments and multivariate models, such as principal component analysis (PCA) or partial least squares (PLS), with the spectra collected. Spectra can also be downloaded from the web to build the models offline. SCiO has another Android/iOS app (‘SCiO’) with a set of pre-established calibration models for measuring nutritional or compositional values of some foods and drugs.

The NeoSpectra Micro Development Kit consists of three tungsten halogen lamps, a monolithic micro-electro-mechanical system (MEMS) Michelson interferometer and a single InGaAs photodetector. The dimensions of the device are 32 × 32 × 22 mm with a weight of 17 g. The NeoSpectra Micro is connected to a Raspberry Pi that acts as a host and allows connection via a universal serial bus (USB) to a laptop. The software (Windows and Linux) allows you to set a limited number of parameters, such as the scan time, run mode (single or continuous) or data interpolation in each spectrum collected. NeoSpectra has to be calibrated each time the software is started. A reflection standard, such as Spectralon^™^ (99% reflectance), is recommended for the calibration. A warm-up time (20 min) is required before taking the measurements. The wavelength range is from 1350 to 2558 nm and the resolution is set to 16 nm (measured at 1550 nm). The acquisition time is around 4–5 s. NeoSpectra is intended for use in contact measurements. The spectral data collected are managed using the supplied software (SpectroMOST) and saved directly to the hard disk in ASCII format.

### 2.2. Samples

Forty-five commercial milks were purchased from supermarkets in Spain, Italy and Switzerland (the composition of the milk can be found in the Supplementary Information). Most samples were cow milks in liquid form, all UHT (Ultra-High-Temperature processing) treated. Samples were chosen covering the maximum range of protein (1.3–7 g/100 mL), fat (0.1–3.7 g/100 mL), and carbohydrate (2.5–13.5 g/100 mL) content available in commercial milk, and as demonstrated in a previous work [33], they span the main variability of commercial milk samples (the analysis of a higher number of samples would not have increased the sample variability). Commercial milk actually has a low degree of variability, as nutritional values are restricted to a limited range. According to European regulation [34], the fat content in skimmed-milk must be less than 0.5% (*m*/*m*), in semi-skimmed milk it must be between 1.5% (*m*/*m*) and 1.8% (*m*/*m*) and in whole milk it cannot be inferior to 3.5% (*m*/*m*). To overcome this limited range (e.g., the highest fat content we could find in supermarkets was 3.7 g/100 mL) and to incorporate more variability, we decided to use milks from three different countries. For this reason, infant formulas and milks with different commercial characteristics (high protein, high carbohydrates, lactose free and fibre-containing), not included in the three groups mentioned above, were also considered.

The compositional information of the labels was taken as reference values to build the multivariate prediction models.

### 2.3. Statistical Data Analysis 

The average of three spectra recorded for each milk sample were used to build the **X** matrix, while the compositional values declared by the producer were used to build the **y** vectors. 

PLS Toolbox 8.8.1 (Eigenvector Inc., Manson, WA, USA) for MATLAB 2020a (Mathworks Inc, Natick, MA, USA) was used for data analysis. Principal component analysis (PCA) and cluster analysis were used for the exploratory and qualitative analysis and partial least squares (PLS) regression for quantitative analysis. Different spectral pre-processing methods were tested: multiplicative scatter correction (MSC), standard normal variate (SNV), as well as first and second Savitzky–Golay derivatives with different numbers of smoothing points (from 7 to 27 points). In the case of the prediction of proteins, orthogonalization [35] was used to improve the results. After spectral pre-processing, the data were mean-centred. Several variable selection methods were applied: variable importance in projection (VIP), interval PLS (iPLS), recursive PLS (rPLS) and selectivity ratio (sRatio). The best model was chosen as the best compromise between the lower root mean square error of cross-validation (RMSECV), the higher r^2^ value of the regression line between the predicted and the measured values (r^2^ corresponds to the correlation coefficient of the regression line) and the smaller number of factors. 

The Venetian blinds method (with 22 data splits and five samples per blind) was used for the cross-validation. For external validation, about 2/3 of the data was selected for the training set and about 1/3 of the data was selected for the test set using the Kennard–Stone algorithm. 

The multivariate limits of detection were calculated using the approximate expression for the sample-specific standard error of prediction (SEP), as derived by Faber and Bro [36].

## 3. Results and Discussion

### 3.1. Optimization of the Instrumental Setup

The milks were stored in a refrigerator (4 °C) after the purchase in local supermarkets (and during the transportation between countries) and left at room temperature before analysis. No thermostatic measurements or other pre-treatments were performed to better simulate rapid routine measurement conditions, although some studies have reported that better results are obtained in multivariate calibration models after pre-treatments such as heating or sonication [33]. Each bottle of milk was gently stirred, and the sample was analysed in triplicate. The average of the three spectra was used to build the multivariate models. Milks were analysed on different days and in random order.

#### 3.1.1. SCiO

For the SCiO measurements, each sample was measured from the top in a silica glass beaker at a fixed distance of 5 mm from the milk (distance was maintained fixing the instrument to a support). Different distances from 1 to 15 mm were tested, and 5 mm was selected as a compromise between a maximum spectroscopic signal (highest reflectance and better signal to noise ratio) and the need for the instrument not to touch the liquid directly. For a given distance, we observed that the orthogonal configuration between the sample and the instruments assured the higher signal (the tilting of the instrument causes a decrease in the signal) and for this reason this configuration was used for all the measurements. Replicates were taken in different parts of the sample, assuring that the experimental conditions did not change. 

Because the instrument is not in direct contact with milk, ambient light conditions may affect the measurements. To check this, and although all measurements were taken in the same laboratory, different precision measurements within the three replicates of a milk (minimum, maximum, mean and median relative standard deviation) were compared in different times during the day as well as on different days, however, no trends or significant differences were observed. 

To test the influence of the experimental session on the instrumental measurements, different PLS models were performed using only the measurements in one session and compared to the global PLS model with all measurements. No significant differences in the RMSECV values of the different models were found. 

The whole analytical process did not take more than 30 s for each milk. 

#### 3.1.2. NeoSpectra

After several tests with the NeoSpectra device, for the final measurements we used a 45 × 22.5 × 12.5 mm quartz cuvette (Hellma, Jena, Germany) that completely covered the NeoSpectra window (20 × 20 mm). The use of a cuvette that totally covers the window facilitates the measurement, as no optimization of the measurement conditions is needed, and there is no influence from the ambient light conditions. The cuvette did not show any appreciable signal in the spectroscopic region considered. Different experiments were performed by coating the backside of the quartz cuvette with reflective materials (such as the aluminium reflector), but the spectra did not change significantly with or without the reflective materials.

After every measurement, the cuvette was carefully washed with water and then rinsed with acetone to remove any residue and dried carefully before the next measurement. Replicates were taken in different parts of the sample, assuring that the experimental conditions did not change.

An attempt was also made to use a quartz cuvette with a narrower optical path (1 mm), but in this case, each measurement was significantly complicated by the difficulty of cleaning the cuvette after each analysis. Since we were interested in optimizing a fast and easy-to-use analytical method, we decided to discard this cuvette.

We also tried to place a drop of milk directly on the NeoSpectra window (without using any cuvette), but we ruled out this option due to the irreproducibility of the results. Several spectra were recorded placing the drop in different parts of the window and variations were observed in terms of signal intensity and noise. This led us to the conclusion that the signal depended heavily on the position of the drop. Moreover, we observed that during the spectroscopic measurement, the window temperature increased greatly due to the heat emitted by the three tungsten halogen lamps, which also affects the measurements. For these reasons, we discarded the direct measurements with a drop of milk on the window and came to the conclusion that the whole window should be covered during the analysis. 

At the beginning of each measurement session an instrumental calibration was performed, which involved a background measurement with a Spectralon^™^ diffuse reflectance standard as required by the instrument. Making a background before each analysis did not improve the performance of the prediction models, nor the quality of the spectra, so a background measurement was made at the beginning of each set of experiments. 

Scanning time was optimized reaching the best compromise at 5 s: less time resulted in more noisy spectra, while higher times did not change the quality of the signal. 

#### 3.1.3. Spectroscopic Signals

**Figure 1:**
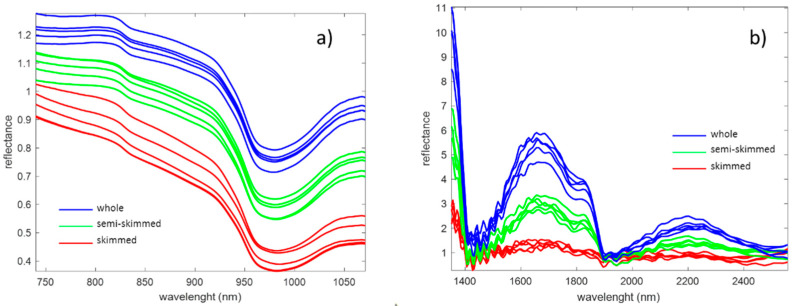
Milk spectra recorded using (**a**) SCiO and (**b**) NeoSpectra.

Figure 1 shows the mean spectra of the analysis of the milk samples with SCiO (Figure 1a) and NeoSpectra (Figure 1b). These average spectra correspond to the raw data produced by the instruments and imported into MATLAB without any prior treatment. SCiO spectra consist of 331 points between 740 and 1070 nm and the NeoSpectra spectra consist of 134 points between 1350 and 2558 nm. Some recorded spectra are shown as examples in Figure 1. The spectra are coloured by the level of fat according to the classification given by the European Regulation (skimmed, semi-skimmed and whole milk as shown in the figure).

Looking at the spectra in Figure 1, it can be seen that the NeoSpectra data are noisier than the SCiO data. The NeoSpectra noise in Figure 1b may be due to the internal reflectance of the optical interface used, although the bands do not appear to follow the regular pattern of a sinusoidal wave superimposed on the signal [37]. Another explanation for the noise may be the heat caused by the three tungsten halogen lamps, as it may affect the signal-to-noise level [38].

Milk is a turbid opaque liquid that heavily scatters light due to the presence of fat globules (basically sized between 1 μm and 10 μm) and protein micelles in suspension (below 200 nm) [19,39,40]. Light scattering in milk has been shown to be largely due to fat globules, with a small contribution from protein micelles [41]. In both Figure 1a,b, low-fat milks have lower reflectance values and high-fat milks have higher reflectance values. This high scattering raises serious concerns about the chemical information that can be extracted from the absorption bands of the spectra. Low reflectance values around 970, 1450, 1950 nm and above 2400 nm were recorded due to the high water absorption [42]. The characteristic absorption bands of the milk components are very weak compared to the water bands and are difficult to visualize, although several absorption bands corresponding to major milk components have been reported in the wavelength range of SCiO and NeoSpectra [42,43].

### 3.2. Multivariate Statistical Analysis

#### 3.2.1. Spectral Pre-Treatment and Exploratory Data Analysis

The best spectral pre-processing for the SCiO data was found to be first-order polynomial smoothing with 15 points, followed by the Savitzky–Golay second derivatives (both for the qualitative and quantitative methods), while the best pre-processing for NeoSpectra was found to be first-order polynomial smoothing with 15 points. 

**Figure 2:**
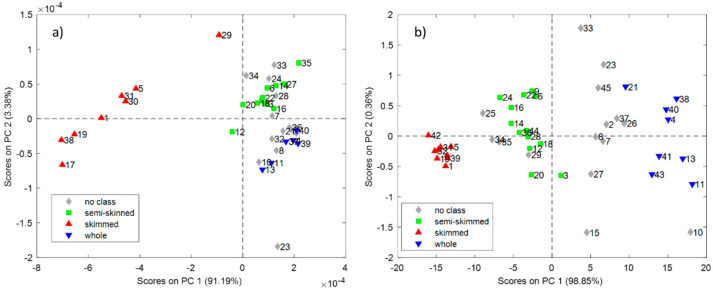
Score plots for the principal component analysis (PCA) models for SCiO (**a**) and NeoSpectra (**b**).

First, a PCA model with milk spectra was calculated. Figure 2 shows the score plots for the first two PCs (Figure 2a shows the SCiO score plot, Figure 2b shows the NeoSpectra score plot). Interestingly, the samples are located in the space according to their level of fat. Since the samples are coloured according to the European regulation [34], there are some milks not classified on the typical ‘skimmed’, ‘semi-skinned’ and ‘whole’ milk levels (these samples are labelled as ‘no class’ in Figure 2a,b. The SCiO model needs two PCs to describe the three groups of fat samples. The first PC mainly describes the difference between low-fat milk and the other groups, while the second PC explains the difference between medium and high-fat milk samples. The NeoSpectra model differentiates, with only one component that accounts for 99% of the information, the milks that differ in their level of fat. No other trends were observed due to other characteristics (amount of protein, amount of carbohydrates, lactose/lactose-free, or country of origin). 

#### 3.2.2. Cluster Analysis

**Figure 3:**
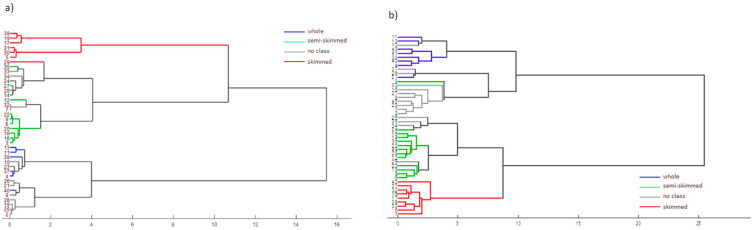
(**a**) Dendrogram for the SCiO fat data and (**b**) the dendrogram for the NeoSpectra fat data.

An agglomerative hierarchical cluster analysis (HCA) was performed to cluster milk samples according to their European classification (Figure 3—it is worthwhile to note that there are milk samples that do not fall into EU regulation groups, and for this reason they are depicted as “no class” in the figure). For SCiO the best results were obtained with the Ward’s method using the autoscaled scores (first two PCs) as the input variables. For NeoSpectra, the best results were obtained with the averaged paired distance using the whole data set as the input variables. 

As we expected, good results were achieved. All the samples with a declared level of fat (skimmed in red, semi skimmed in green, whole in blue), except sample 29 (a skimmed milk grouped in the semi-skimmed milk cluster), were correctly clustered in the SCiO dendrogram. A possible explanation of this misclassification is due to its protein content, which shows the maximum value of 7.0 g/100 mL. This feature probably affects the scattering properties of this sample (due to the scattering caused by the protein micelles), making its spectra more similar to those in the semi-skimmed category. Samples without European classification are clustered according to their fat content. 

#### 3.2.3. Prediction of Fats and Proteins

##### Prediction of the Fat Content

**Figure 4:**
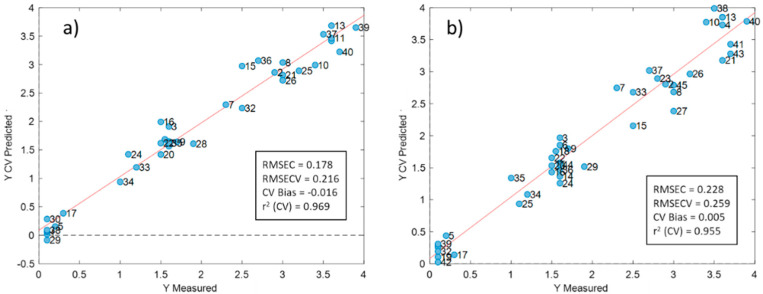
Regression line between the measured and predicted fat content for SCiO (**a**) and NeoSpectra (**b**).

**Figure 5:**
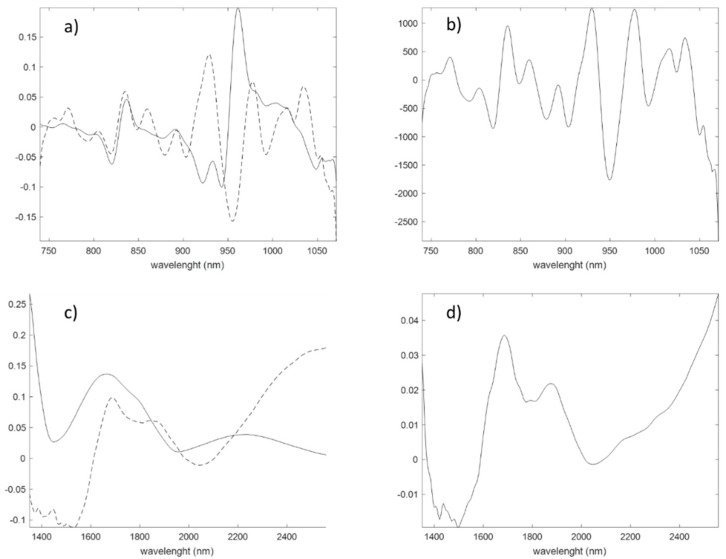
Loadings and regression coefficients of the PLS models. SCiO: (**a**) first (solid line) and second (dashed line) loadings; (**b**) regression coefficients. NeoSpectra: (**c**) first (solid line) and second (dashed line) loadings; and (**d**) regression coefficients.

Figure 4a shows the prediction results of the best PLS regression models built with SCiO data and cross-validated (as described in Section 2.2). Very good results were achieved: the root mean square error of cross-validation (RMSECV) was 0.216 g/100 mL, with an r^2^ between predicted and measured values of 0.969 for the best model combining the minimum number of factors and a good fit between the predicted and measured values (indicated with * in Table 1). Two factors were needed to explain 97.82% of the *y*-variance. Sample 23 was detected as an outlier and was thus removed. This sample contained added cereals (as declared by the producer) and that is why its spectrum was quite different from the others. Smoothing followed by Savitzky–Golay first-derivatives and mean centring provided the lowest RMSECV, but for a more complex model (using five factors) and for this reason discarded. Using MSC and SNV to pre-process the spectra did not improve but even worsened the prediction results, as reported in Table 1, which shows the best results obtained for the main pre-treatments used. These results seem to highlight the importance of light scattering in prediction. Despite the importance of light scattering, absorption is probably not negligible, as is shown in the loading plots (Figure 5a) around 930 nm (third overtone C-H stretch vibration of triglycerides). Figure 5b shows the regression coefficients of the SCiO PLS model.

We also built a PLS model using the SCiO lab website. On this website, the algorithm is proprietary and therefore not accessible to the users. The model was cross-validated with Venetian blinds (five samples per blind), and no values of root mean square error of calibration (RMSEC) bias nor regression coefficients were provided. There were also no plots for the selection of factors nor the removal of outliers (this is done automatically). The only plot provided is the predicted versus measured values. Four factors were automatically selected, with a RMSECV of 0.272 g/100 mL and an r^2^ between the predicted and the measured values of 0.950. This PLS model provided worse RMSECV and r^2^ values and used a higher number of factors than the best model obtained for SCiO (Table 1).

Satisfactory results were also obtained with NeoSpectra data, with a slightly higher RMSECV value for the best PLS model obtained. The prediction results are shown in Figure 4b for a two-factor model. The RMSECV is 0.259 g/100 mL, with an r^2^ between the predicted and the measured values of 0.955. As in the case of SCiO, the loadings of the PLS NeoSpectra model (Figure 5c) have the maximum value at one of the fat-related wavelengths (1690, first overtone of CH_3_ stretching). Other important fat-related wavelengths seem less important: 1722 nm (first overtone of CH_2_ antisymmetric stretching), 1754 nm (first overtone of CH_2_ symmetric stretching), 2302 nm (combination of CH_2_ asymmetric stretching and bending vibrations) and 2340 nm (combination of CH_2_ symmetric stretching and bending vibrations) [42,43]. Neither MSC nor SNV improved the prediction results and on the contrary, worsened the prediction performance, as reported in Table 1. 

The presence of systematic errors in the SCiO and NeoSpectra PLS models was checked by comparing the intercept of the regression lines in Figure 4a,b with the theoretical value of 0. In both cases, the theoretical value of 0 was included in the confidence interval for the intercept ($b_{0}\pm t_{\alpha ,degrees\;of\;freedom}\cdot s_{b_{0}}$), showing the absence of systematic errors (Table 2).

**Figure 6:**
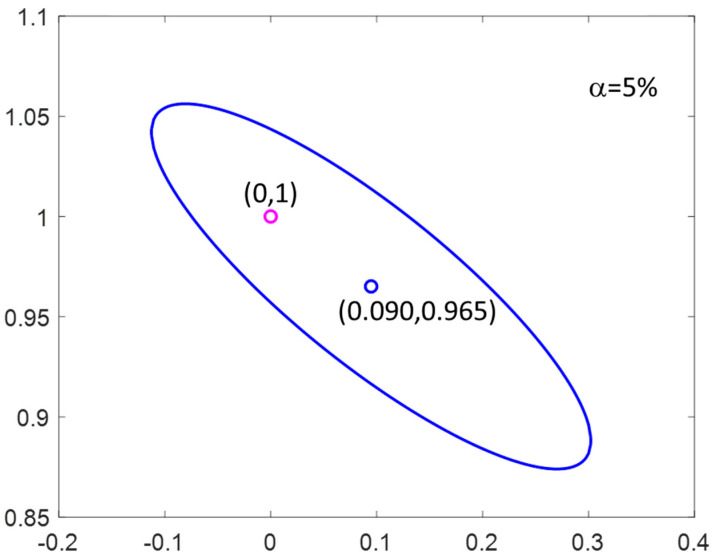
Joint confidence interval for the intercept and the slope of the comparison between the SCiO and NeoSpectra PLS best predictions.

Since we analysed the same milks with SCiO and NeoSpectra, to compare their fat predictions obtained with the two optimal PLS models (indicated with * in Table 1), we used a linear approach with the joint confidence interval for the intercept and the slope [44]. The theoretical regression line obtained with the prediction of the two instruments should have intercept = 0 and slope = 1 if the predictions of the two PLS models were identical. Therefore, if the two sets of predictions provide comparable results, the joint confidence interval of the intercept and the slope of the regression line between the two methods has to include the theoretical point (0, 1). Figure 6 shows that there is no statistically significant difference between the two sets of predictions at a significance level α= 5%.

The calculation of the multivariate limit of detection was estimated according to the following expression [36]:

(1) $$\mathrm{LOD}=\Delta \left(\alpha ,\beta ,\upsilon \right)\cdot \mathrm{RMSEC}\cdot \sqrt{1+h_{0}}$$

The RMSEC is obtained from the SCiO and NeoSpectra PLS best models (indicated as * in Table 1). The leverage *h*_0_ quantifies the distance of the predicted sample at zero concentration to the mean of the calibration set [45]. We estimated *h*_0_ as the average of the leverage values corresponding to the samples with the lowest concentration of fat. The term Δ(α,β,υ) takes into account the α and β probabilities of wrongly concluding the presence/absence of analyte. From a practical point of view, and when the number of degrees of freedom υ is high (υ≥ 25), Δ(α,β,υ) can safely be approached to 2. The LOD estimated for the PLS SCiO model was 0.376 g/100 mL and the LOD for the PLS NeoSpectra model was 0.468 g/100 mL.

With these limits of detection, other milks than skimmed milks can be quantified, while skimmed milks lie below the limit of detection. The LOD can then be used as a threshold between skimmed milk and the rest of the samples. If the prediction of fats in a milk sample is below the LOD, it is possible to qualitatively state that the sample is a skimmed milk. If the predicted value is above the LOD, it will be possible to quantify it exactly (considering the associated prediction error).

In addition to using cross-validation to validate the PLS models, we also split the data into a training set (about 2/3 of the data) and a test set (about 1/3 of the data), using the Kennard–Stone algorithm to perform an external validation and comparing the results with the one obtained by cross-validation. Table 3 shows the results of the models built for SCiO and NeoSpectra, comparing in each case the root mean square error of calibration (RMSEC) and the root mean square error of cross-validation (RMSECV) for the models built with the whole set of samples, and the root mean square error of prediction (RMSEP) found when predicting the test set with the PLS model built using the training set. The results in Table 3 are comparable to the results obtained using all the samples to build the PLS models, proving the robustness of the models.

The RMSEP for the SCiO model built with Kennard–Stone samples is lower than the respective RMSECV value (not in the case of the NeoSpectra model). It is worth remembering that the Kennard–Stone algorithm chooses the samples to better represent the multivariate space of the data. Because of this, the samples with extreme characteristics were selected in the calibration set to better describe the multivariate space, improving the chances of obtaining a smaller error in the test set as more typical samples are included [32]. 

Our prediction errors are higher than other reported values for the prediction of fats in milk from farms over a similar NIR wavelength range in reflection mode (e.g., RMSECV of 0.098% fat *wt*/*wt* but with eight factors [42]). However, it is important to note that in our case, we did not perform any pre-treatment of the milk or use any special measuring head for the samples, apart from using portable low-cost instrumentation. It is also important to mention that we used the compositional information in the labels as the reference values for the PLS models, which may deteriorate the prediction ability of our models. European regulation about nutrient values declared in food labels [46] states that for declaration on foods other than food supplements, the tolerance for fat when the content in fat is lower to 10% is ±1.5 g. Therefore, a significant part of the RMSECV of the PLS models found with SCiO and NeoSpectra data may be due to the allowed tolerance values declared in the milk labels. A recent article [33] reported the prediction of fat in milk samples using a portable ATR -FT-MIR (Attenuated total reflection-FT-MIR) spectrophotometer, with prediction values of 0.31 g/100 mL using one PLS factor. We therefore consider that these are good prediction values for on-site rapid and simple quality control. It is worthwhile to note that the number of samples is adequate according to ASTM E1655-17 [47], which indicates that the minimum number of samples for the calibration set must be equal to 6 (*k* + 1) for the mean-centred data (where *k* is the number of latent variables) and the prediction set must be equal to 4*k* (see e.g., [48]).

##### Prediction of the Protein Content

The best model for SCiO was obtained with first-order polynomial smoothing with 15 points followed by the Savitzky–Golay second derivative and final mean centring as spectral data pre-treatment. 

The best PLS model for SCiO data was built with six factors and explained 89.68% of the information in **y**. This number of factors is significantly higher than the number of factors used for the prediction of fats, which made us think about the existence of some hidden effects, such as the dependence of the fat level on the prediction of proteins. This would not be surprising since stable fat globules are surrounded by a membrane formed by the protein molecule [41]. It is also worth noting that the protein values are not as homogeneously distributed, as in the case of fats, since a high percentage of commercial milks have protein contents between 3 and 4 mg/100 mL.

We performed variable selection in this model (iPLS method), and 12 of the initial 331 SCiO variables were selected. A satisfactory RMSECV value of 0.393 g/100 mL with an r^2^ between the predicted and the measured values of 0.863 were obtained. Sample 23 was also removed in this model. 

**Figure 7:**
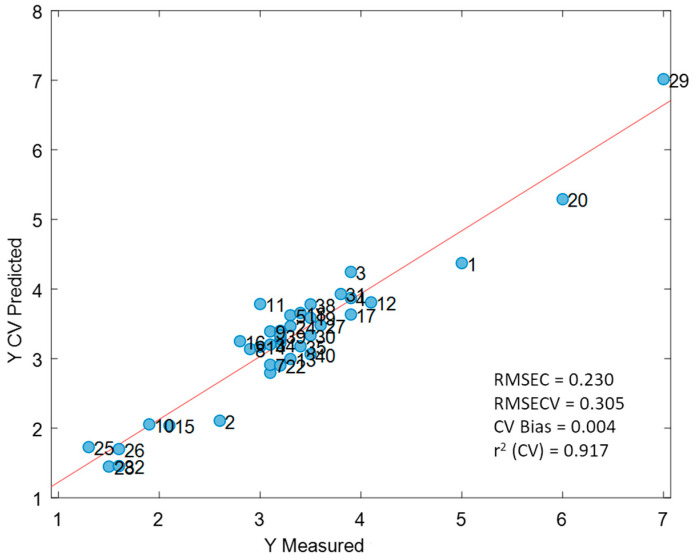
Regression line between the measured and predicted protein contents for SCiO.

As a way to improve the prediction of proteins, we tried to subtract the information explained only by the fat content by orthogonalizing the spectra with respect to the subspace spanned by the fat content, following the procedure described in [35]. In brief, this method requires the spectra of the samples to be measured under the different conditions that modify the spectra (in this case, the different fat concentration). These variations are not related to the concentration of proteins and can therefore be removed with an orthogonalization step before modelling. The most appropriate samples for orthogonalization (after several trials) were the samples with protein values between 3.1 and 3.3 g/100 mL, which cover the range of values for fat concentration. After orthogonalization and variable selection (iPLS method, 26 wavelengths selected), the PLS model provided better results, with a RMSECV value of 0.305 g/100 mL and an r^2^ between the predicted and the measured values of 0.917 (Figure 7). 

The estimated LOD for this method is 0.655 g/100 mL protein content, so the method is able to quantify all the milk samples (taking into account the associated prediction error). It is difficult to remove all the contribution due to fat content. Most likely, also in this case, we are using some of the scattering information to predict the protein content in milk samples, along with some chemical information. 

It is worth noting that the use of the compositional information in labels as reference values of the PLS models, also for protein prediction, can deteriorate the predictive ability of the models, as explained in the case of fat prediction. 

**Figure 8:**
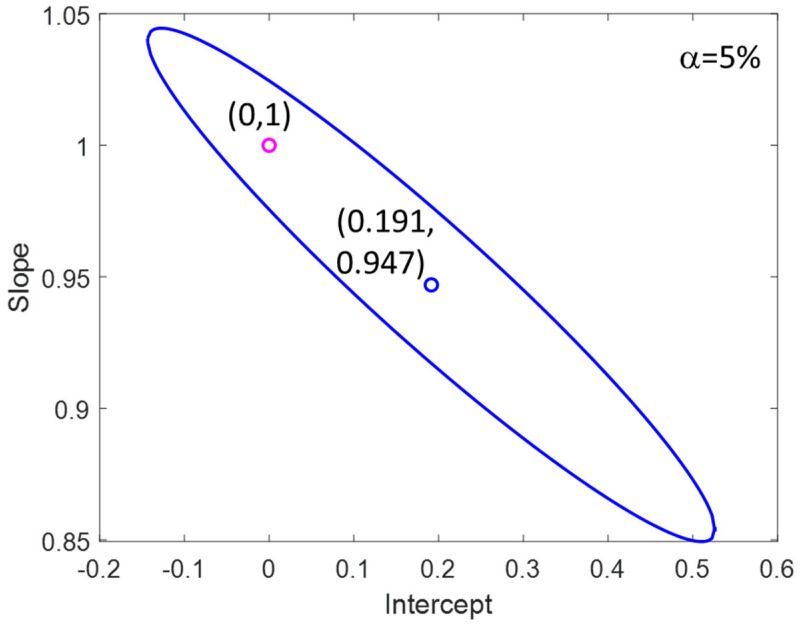
Joint confidence interval for the intercept and the slope of the comparison between the declared and predicted % of proteins.

Figure 8 shows the joint confidence interval for the intercept and the slope comparing the declared and the predicted values of the protein in milk. From the figure, we can conclude that there are no statistical differences between the declared and the predicted values along the linear range. The absence of constant errors was also verified, as the confidence interval for the intercept (0.191 ± 0.258) is not statistically different from 0 for a level of 5%.

For the validation of the PLS models, we also split the data into a training set (about 2/3 of the data) and a test set (about 1/3 of the data) using the Kennard–Stone algorithm to perform an external validation, and compared the results with the one obtained by cross-validation. The root mean square error of prediction (RMSEP) value was 0.290 g/100 mL with an r^2^ between the predicted and the measured values of 0.883. The results are comparable to the results obtained using all the samples to build the PLS models, proving the robustness of the models. The r^2^ value for the test is set as slightly worse than for the overall set of samples, because the selected range of protein concentrations in the test set is narrower than for the whole set of samples, making it more difficult to obtain a good fit between the predicted and the measured values.

The PLS model using the NeoSpectra data did not provide good results, with an RMSECV value close to 1 g/100 mL and r^2^ values between the predicted and the measured values around 0. Variable selection did not improve the prediction ability, nor data pre-treatments such as orthogonalization. A possible explanation for the poor prediction of the protein content with NeoSpectra may come from the size of the protein. Protein micelles, which are smaller than milk fat globules, are approximately spherical colloidal particles with an average diameter of about 200 nm, but ranging from 50 to 600 nm in diameter [19,39,40]. Since the wavelengths used by SCiO are shorter than those used by NeoSpectra, this may ease the dispersion of light by the protein micelles.

Since we saw that the samples are clustered according to their fat level (Figure 2 and Figure 3), an attempt was made to improve the prediction of proteins with SCiO by making different PLS models according to the fat level. In addition to making an individual PLS model for skimmed, semi-skimmed and whole milk, we also tried to group the semi-skimmed and whole milks, as these two groups partially overlap (Figure 2). The only subgroup that improved the results of the global PLS model for protein with all samples was skimmed milk, with a three-factor PLS model after variable selection (iPLS method, six wavelengths selected), which explains 99.34% of the information in *y* and has an RMSECV value of 0.265 g/100 mL. It is worthwhile to note that, in the case of protein content prediction, a higher number of samples should have been analysed to obtain statistically significant results (due to the high number of latent variables necessary to predict the property). Nevertheless, we consider these results promising and worthy to be reported. 

##### Prediction of Carbohydrates Content

As expected, and since carbohydrates dissolve primarily in milk without forming globular aggregates of micelles, their detection in a system mainly based on light scattering was not as accurate as would be desirable. With the SCiO data, a PLS model was obtained using four factors with an RMSECV value of 0.639 g/100 mL and an r^2^ value of 0.883. iPLS was used as the variable selection method and 47 wavelengths were selected.

Similar to protein prediction, the PLS model for carbohydrates from NeoSpectra data did not provide good results. Variable selection did not improve the prediction ability.

PLS discriminant analysis (PLS-DA) was applied to predict the presence or absence of lactose. A dummy vector **y** was created by assigning a 0 to lactose-free milk (as declared by the producer) and a 1 to lactose-containing milks. After variable selection (the iPLS method selected 11 variables), the model with SCiO data (five factors) could correctly classify 38 out of the 40 milk samples that included information about lactose. The sensitivity and specificity classification results for the two classes are shown in Table 4.

For the validation of the PLS-DA model we also split the data into a training set (about 2/3 of the data) and a test set (about 1/3 of the data) using the Kennard-Stone algorithm to perform an external. The sensitivity and specificity for the test was 1.0 in both cases, showing the validity of the PLS-DA model. As in the case of the protein content prediction, a higher number of samples should have been analysed (due to the high number of latent variables necessary to predict the property). Anyway, we also considered these results very promising and worthy to be commented.

The model with NeoSpectra data did not provide good predictions for the presence/absence of lactose.

#### 3.2.4. Data Fusion

**Figure 9:**
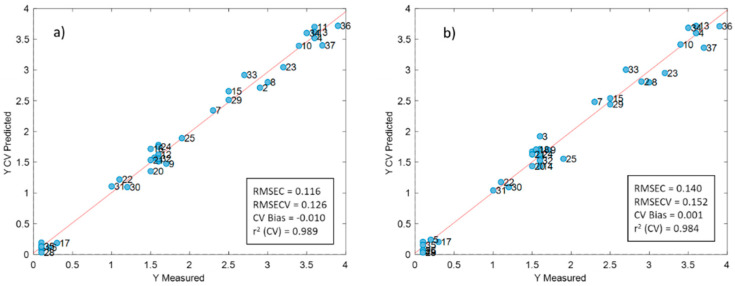
Results of the low-(**a**) and mid-(**b**) level data fusion models for the prediction of fats.

Since SCiO and NeoSpectra use different wavelength ranges, we considered fusing the data from the two sensors (low- and mid- level data fusion), to try to improve the prediction ability of the models. In the low-level data fusion, we combined the pre-treated data from the two instruments and we built a PLS model after autoscaling the data. The resulting model provided slightly better results than those of the two individual PLS models: two latent variables explained 98.06% of the information in *y*, with an RMSECV value of 0.197 g/100 mL and an r^2^ between the predicted and the measured values of 0.973. Both instruments contributed to a similar degree to the model loadings, demonstrating that both instruments provided complementary data. Variable selection was carried out in this model; the iPLS method selected 19 variables, most of them from SCiO. This model provided the best results obtained in this research. The optimal PLS model used two latent variables that explained 99.08% of the information in *y*, an RMSECV value of 0.126 g/100 mL and an r^2^ between the predicted and the measured values of 0.989 (Figure 9a). With this model, the estimated LOD for fat was 0.245 g/100 mL. 

In the mid-level data fusion, we performed two separate PCA models (one for each instrument) and we put together the scores of the two first PCs for each instrument. We then built a PLS model by autoscaling the data. The model also provided better results than those of the two individual prediction models. Two latent variables explained 98.66% of the information in *y*, with an RMSECV of 0.152 g/100 mL and an r^2^ between the predicted and the measured values of 0.984 (Figure 9b). With this latter model, the estimated LOD for fat was 0.295 g/100 mL. A part of the skimmed milks can be thus quantified. 

External validation was also performed, as previously described using the Kennard–Stone algorithm (training set about 2/3 of the data, test set about 1/3 of the data) obtaining the very promising results of RMSEP = 0.132 g/100 mL with an r^2^ = 0.989 for the low level data fusion, and RMSEP = 0.133 g/100 mL with an r^2^ = 0.989 for the mid-level data fusion. In addition, in this case the number of samples is adequate according to ASTM E1655-17.

## 4. Conclusions

In this manuscript, we described the optimization of two miniaturized low-cost spectrophotometers to the analysis of commercial milk samples. 

Both SCiO and NeoSpectra can provide a rapid and reliable analysis of fat in commercial milk (in the range 0.1–3.7%) without any sample pre-treatment. Cluster analysis can correctly cluster milk according to their fat level. Semi-skimmed and whole milk fat values were correctly predicted, while part of the skimmed milk fat values lie below the limit of detection of both models. When coupling the data collected by both instruments (in a data fusion approach), the results were even better. 

The results suggested that both devices have a potential to be used e.g., in quality control phases in the milk production. 

SCiO can also predict protein content (the LOD of the model allows predicting all the protein values) and the presence/absence of lactose in commercial milk samples with a high degree of accuracy. SCiO, therefore, showed the best performance. The SCiO also has the advantage that you do not need to it to any computer, you only need a smartphone app.

In our opinion, this research leads to another significant finding. 

The development and use of affordable and portable instrumentation go in the line of green analytical chemistry (which involves a drastic reduction of costs, analytical steps, sample pre-treatment, energy and reagents), and for this reason, the use of these devices should be promoted. 

Nevertheless, the best operational conditions must be optimized to assure the best quality signal and therefore, to obtain the best predictions, as it is shown in the paper. This often involves the use of chemometric methods, which sometimes are not so straightforward (e.g., orthogonalization for the protein models) to analyse the data. This point is obvious to a researcher, but it is not so obvious for a non-expert user, who may not make the most of these instruments. Consumer-oriented companies that advertise these instruments as appropriate tools for everyday applications by non-expert users should consider these issues. 

## Figures and Tables

**Table 1:** Comparison of the best partial least squares (PLS) models obtained with different pre-treatments for the prediction of fat. Errors are expressed as % fat (g/100 mL). Apart from the listed pre-treatments, all the models were finally mean centred. Best models are indicated with *. LVs= Latent Variables

		SCiO (740–1070 nm)	NeoSpectra (1350–2558 nm)
Smoothing	RMSEC	0.182	0.228
	RMSECV	0.226	0.259 *
	r^2^ CV	0.965	0.955
	LVs	5	2
Smoothing + SNV	RMSEC	0.167	0.359
	RMSECV	0.234	0.481
	r^2^ CV	0.962	0.847
	LVs	6	3
Smoothing + MSC	RMSEC	0.156	0.363
	RMSECV	0.223	0.494
	r^2^ CV	0.966	0.840
	LVs	6	3
Smoothing + 1st SG	RMSEC	0.150	0.210
	RMSECV	0.196	0.267
	r^2^ CV	0.974	0.952
	LVs	5	3
Smoothing + 2nd SG	RMSEC	0.178	0.217
	RMSECV	0.216 *	0.288
	r^2^ CV	0.969	0.944
	LVs	2	-
SCiO lab	RMSEC	-	-
	RMSECV	0.272	-
	r^2^ CV	0.950	-
	LVs	4	-

**Table 2:** Confidence interval for the intercept showing the absence of systematic errors in both PLS models.

	$b_{0}$	$s_{b_{0}}$	Lower Confidence Interval	Upper Confidence Interval
SCiO	0.057	0.078	−0.101	0.215
NeoSpectra	0.068	0.064	−0.062	0.198

**Table 3:** PLS models for fat prediction with the training and test sets.

		SCiO (2 Factors)	NeoSpectra (2 Factors)
Training set	RMSEC	0.178	0.228
	r^2^ CALIBRATION	0.978	0.965
	RMSECV	0.216	0.259
	r^2^ CV	0.969	0.955
Test set	RMSEP	0.176	0.287
	r^2^ PREDICTION	0.981	0.980

**Table 4:** PLS-DA results for the presence or absence of lactose using the SCiO data.

	No Lactose	With Lactose
Sensitivity (calibration)	1.0	0.9
Specificity (calibration)	0.9	1.0
Sensitivity (cross-validation)	1.0	0.9
Specificity (cross-validation)	0.9	1.0

## Supplementary Materials

The following are available online at https://www.mdpi.com/2304-8158/9/8/1090/s1, Table S1: List of milks.
