# Supplementary material for: Rapid Analysis of Milk Using Low-Cost Pocket-Size NIR Spectrometers and Multivariate Analysis
Source: Foods. 2020 Aug 10;9(8):1090. doi: 10.3390/foods9081090 (PMC7465951; doi:10.3390/foods9081090)
Supplement: Supplementary file 1 [file foods-09-01090-s001.pdf]

# Rapid analysis of milk using low-cost pocket-size NIR instrumentation and multivariate analysis

Jordi Riu[a], Giulia Gorla[b], Dib Chakif[a], Ricard Boqué[a], Barbara Giussani[b]

[a] Department of Analytical Chemistry and Organic Chemistry, Universitat Rovira i Virgili, 43007 Tarragona, Catalonia-Spain

[b] Dipartimento di Scienza e Alta Tecnologia. Università degli Studi dell'Insubria. Via Valleggio, 9. 22100 Como, Italy

**Supplementary Information**

**Table S1. List of milks**

| Commercial brand | Country | % fat | % protein | % carbohydrates |
|---|---|---|---|---|
| **Asturiana** | Spain | 0.1 | 5 | 4.7 |
| **Puleva** | Spain | 2.9 | 2.6 | 5.3 |
| **Puleva** | Spain | 1.6 | 3.9 | 5.1 |
| **Carrefour** | Spain | 3.6 | 3.9 | 4.7 |
| **Celta** | Spain | 0.2 | 3.3 | 4.8 |
| **Puleva** | Spain | 1.6 | 3.1 | 4.8 |
| **Puleva** | Spain | 2.3 | 3.1 | 5.2 |
| **Hacendado** | Spain | 3 | 2.9 | 7 |
| **Flora** | Spain | 1.7 | 3.1 | 4.8 |
| **Nestle** | Spain | 3.4 | 1.9 | 7.2 |
| **Puleva** | Spain | 3.6 | 3 | 4.7 |
| **Puleva** | Spain | 1.6 | 4.1 | 5.8 |
| **Asana** | Spain | 3.6 | 3.3 | 4.8 |
| **Pascual** | Spain | 1.6 | 3 | 4.9 |
| **Nestle** | Spain | 2.5 | 2.1 | 9.3 |
| **Asturiana** | Spain | 1.5 | 2.8 | 4.6 |
| **Pascual** | Spain | 0.3 | 3.9 | 5.6 |
| **Asana** | Spain | 1.6 | 3.4 | 4.9 |
| **Asana** | Spain | 0.1 | 3.5 | 5 |

| | | | | |
|---|---|---|---|---|
| **Hacendado** | Spain | 1.5 | 6.0 | 4.6 |
| **Pascual** | Spain | 3.6 | 3.9 | 5.7 |
| **Carrefour** | Spain | 1.5 | 3.2 | 4.7 |
| **Nestle** | Spain | 2.8 | 2.9 | 13.5 |
| **Sterilgarda** | Italy | 1.1 | 3.3 | 5.0 |
| **Nestlé** | Switzerland | 3.2 | 1.3 | 8.2 |
| **Nestlé** | Switzerland | 3.0 | 1.6 | 9.1 |
| **Mila** | Italy | 1.6 | 3.6 | 4.9 |
| **Mellin** | Italy | 1.9 | 1.5 | 6.3 |
| **Emmi** | Switzerland | 0.1 | 7.0 | 2.5 |
| **Léger** | Switzerland | 0.1 | 3.5 | 4.5 |
| **Granarolo** | Italy | 0.1 | 3.8 | 3.4 |
| **Plasmon** | Italy | 2.5 | 1.6 | 8.3 |
| **Carrefour** | Spain | 1.6 | 3.1 | 4.8 |
| **Parmalat** | Italy | 1.2 | 3.2 | 4.8 |
| **Accadi** | Italy | 1.0 | 3.1 | 3.1 |
| **Esselunga** | Italy | 1.6 | 3.4 | 5.2 |
| **Coop** | Switzerland | 2.7 | 3.2 | 4.9 |
| **Coop** | Switzerland | 3.5 | 3.2 | 4.9 |
| **Esselunga** | Italy | 0.1 | 3.5 | 5.3 |
| **Coop** | Switzerland | 3.9 | 3.2 | 4.9 |
| **Valflora** | Switzerland | 3.7 | 3.5 | 5.0 |
| **Granarolo** | Italy | 0.1 | 3.2 | 5 |
| **Esselunga** | Italy | 3.7 | 3.4 | 5.1 |
| **Granarolo** | Italy | 1.6 | 3.2 | 5 |
| **Parmalat** | Italy | 3 | 3.2 | 4.9 |
